# Influence of Right Ventricular Dysfunction on Outcomes of Left Ventricular Non-compaction Cardiomyopathy

**DOI:** 10.3389/fcvm.2022.816404

**Published:** 2022-01-31

**Authors:** Wuwan Wang, Wei Chen, Xue Lin, Ligang Fang

**Affiliations:** Department of Cardiology, State Key Laboratory of Complex Severe and Rare Diseases, Peking Union Medical College Hospital, Chinese Academy of Medical Science and Peking Union Medical College, Beijing, China

**Keywords:** right ventricular dysfunction, left ventricular non-compaction, mortality, rehospitalization, strain

## Abstract

**Background:**

Various adverse outcomes such as mortality and rehospitalization are associated with left ventricular non-compaction (LVNC). Due to data limitations, prospective risk assessment for LVNC remains challenging. This study aimed to investigate the influence of right ventricular (RV) dysfunction on the clinical outcomes of patients with LVNC through accurate and comprehensive measurements of RV function.

**Methods and Results:**

Overall, 117 patients with LVNC (47.6 ± 18.3 years, 34.2% male) were enrolled, including 53 (45.3%) and 64 (54.7%) patients with and without RV dysfunction, respectively. RV dysfunction was defined as meeting any two of the following criteria: (i) tricuspid annular systolic excursions <17 mm, (ii) tricuspid S′ velocity <10 cm/s, and (iii) RV fractional area change (FAC) <35%. The proportion of biventricular involvement was significantly higher in patients with RV dysfunction than in controls (*p* = 0.0155). After a follow-up period of 69.0 [33.5, 96.0] months, 18 (15.4%) patients reached the primary endpoint (all-cause mortality), with 14 (26.4%) and 4 (6.3%) from the RV dysfunction group and normal RV function group, respectively. The Kaplan–Meier method and log-rank test revealed that patients with RV dysfunction had a higher risk of all-cause mortality than those in the control group (hazard ratio [HR]: 5.132 [2.003, 13.15], *p* = 0.0013). Similar results were obtained for patients with left ventricular ejection fraction (LVEF) <50% [HR, 6.582; 95% confidence interval (CI), 2.045–21.19; *p* = 0.0367]. The relationship between RV dysfunction and heart failure rehospitalization and implantation of implantable cardioverter-defibrillator (ICD)/cardiac resynchronization therapy (CRT) was not statistically significant (both *p* > 0.05). The multivariable Cox proportional hazard modeling analysis showed that RV dysfunction (HR: 4.950 [1.378, 17.783], *p* = 0.014) and impaired RV global longitudinal strain (RVGLS) (HR: 1.103 [1.004, 1.212], *p* = 0.041) were independent predictors of mortality rather than increased RV end-diastolic area and decreased LVEF (both *p* > 0.05).

**Conclusions:**

RV dysfunction is associated with the prognosis of patients with LVNC.

## Introduction

Left ventricular non-compaction (LVNC) is a rare cardiomyopathy that is characterized by a thin and compacted epicardial layer, trabeculae, and deep intertrabecular recesses in the left ventricular myocardium (1). It is associated with asymptomatic, embolic events, and an inherent risk of malignant arrhythmia. Furthermore, sudden death caused by LVNC can be prevented by inserting an implantable cardioverter-defibrillator (ICD) (2, 3). The increased awareness of LVNC among cardiologists and improved imaging technologies have led to a better understanding of this condition, resulting to it being a widely recognized cardiomyopathy (4). Prospective risk assessment of LNVC is difficult because of the wide variation in its clinical outcomes (5–7). In addition, only a few studies have evaluated prognostic predictors (8).

Right ventricular (RV) dysfunction occurs in a substantial proportion of patients with LVNC (9–11). An accurate and reproducible assessment of RV function is required to assess the prognosis of patients; however, such an assessment remains difficult because of limited data on patients with LVNC, the complex shape of the RV, and a high load dependency. Conventional parameters assessing RV function include tricuspid annular plane systolic excursion (TAPSE), RV fractional area change (FAC), and tricuspid S′ velocity (12). Myocardial functional dynamics can be assessed with good accuracy using a 2D strain imaging technique, such as speckle-tracking echocardiography (STE) (13). Its applicability has been extended to RV function assessments (14) to detect early systolic functional abnormalities during the preclinical stage (15, 16). A recent study evaluated the role of RV function in the clinical outcomes of LVNC and showed that the right ventricular end-diastolic area (RVEDA) index is a strong prognostic marker that independently predicts death or the need for heart transplantation in patients with LVNC and indicates the prognostic value of the RV size (6). However, the prognostic value of factors such as TAPSE and RV FAC remain weak. Moreover, another study on 14 patients with LVNC demonstrated that RV dysfunction is a marker of advanced LVNC and poor prognosis (11); however, its sample size was relatively small. Considering the limited data on RV function with prognostic values of LVNC, the current study aimed to investigate the impact of RV dysfunction on LVNC-related clinical outcomes by accurate and comprehensive measurement of RV function.

## Methods

### Data Source and Study Population

**Figure 1:**
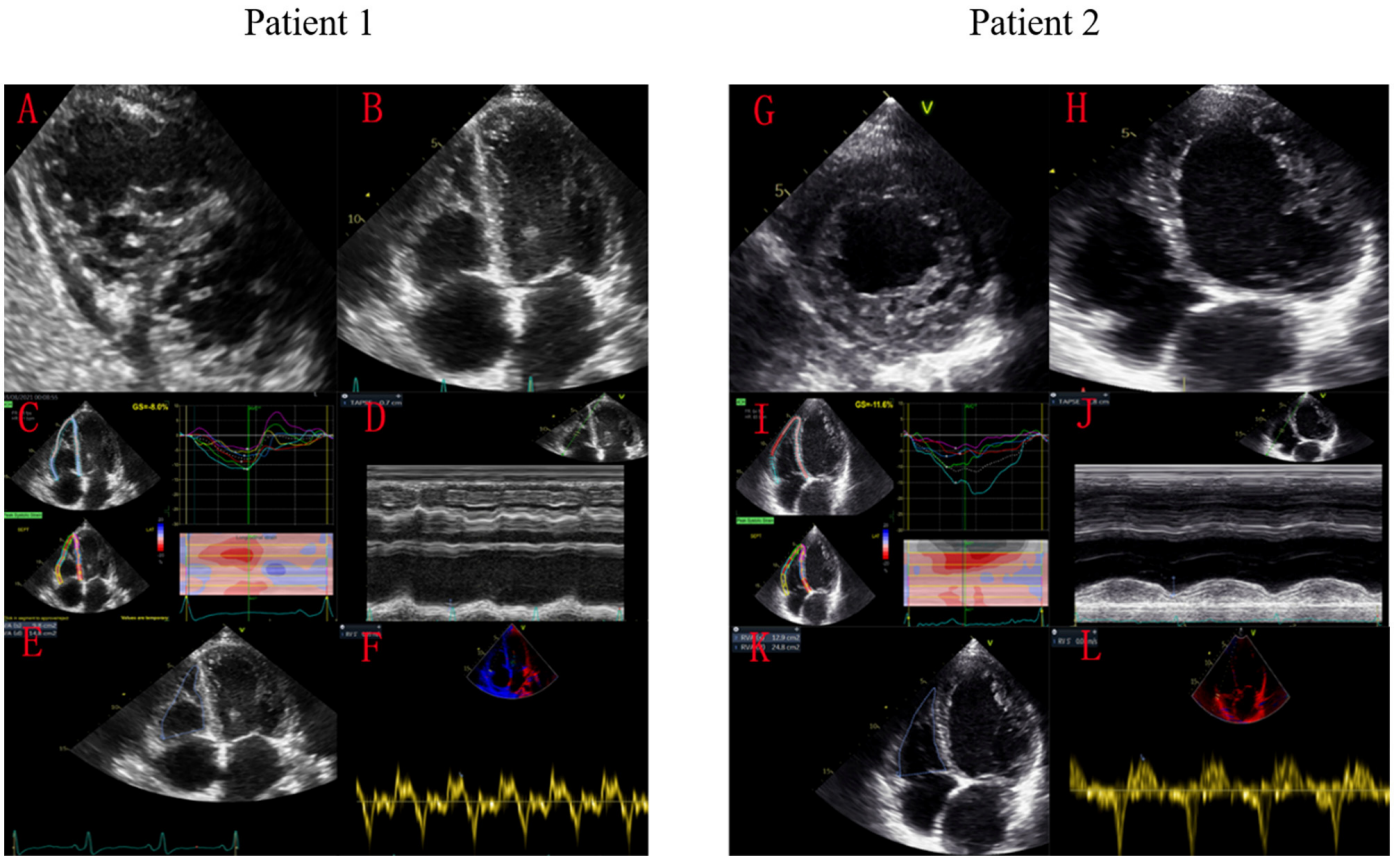
Speckle-tracking echocardiography images of two patients with LVNC and the same LVGLS. Patient 1 with significant RV dysfunction died of cardiogenic shock, while patient 2 survived for long-term after treatment. Patient 1 had significant echocardiographic manifestations of RV non-compaction **(A,B)**. His RVGLS was −8.0% **(C)**, TAPSE was 7mm **(D)**, RV FAC was 24% **(E)**, and tricuspid S′ was 0.05 m/s **(F)**. Patient 2 only had LV echocardiography manifestations of ventricular non-compaction **(G,H)**. His RVGLS was −11.6% **(I)**, TAPSE was 18 mm **(J)**, RV FAC was 47% **(K)**, and tricuspid S′ was 0.07 m/s **(L)**.

Patients diagnosed with LVNC at the Peking Union Medical College Hospital based on the criteria described by Jenni (17) (a non-compacted/compacted ratio >2.0 in end-systole) and had at least one transthoracic echocardiography (TTE) at baseline between January 1, 2006, to June 30, 2021, were enrolled in the study. Patients with other cardiovascular conditions, including ischemic cardiomyopathy, primary valvular illness, congenital heart disease, cancer, or severe multi-system failure, and those who could not complete the follow-up period were excluded. This study was approved by the local ethics committee, and informed consent was obtained from all participants. The patients were categorized into two groups according to RV function, with RV dysfunction defined as meeting any two of the following criteria: (i) TAPSE <17 mm, (ii) tricuspid S′ velocity <10 cm/s, and (iii) RV FAC <35% (Figure 1) (12, 18).

### Baseline Characteristics and Echocardiography

The clinical and demographic characteristics of the patients were collected from chart reviews, laboratory data, and auxiliary examinations at the time of enrollment. Baseline data, including age, sex, systolic blood pressure (SBP), diastolic blood pressure (DBP), heart rate (HR), and the levels of N-terminal fragment of pro-hormone brain natriuretic peptide (NT-proBNP), cardiac troponin I (cTnI), albumin (Alb), hemoglobin (Hb), and creatinine (Cr) were collected. The estimated glomerular filtration rate (eGFR) was calculated according to the Chronic Kidney Disease Epidemiology Collaboration (CKD-EPI) equation (19). Transthoracic echocardiography was performed using commercially available equipment (Vivid 7 and Vivid E9, GE Medical Systems, Horten, Norway). Right ventricular involvement was diagnosed based on the criteria described by Jenni et al. applied to the right ventricle (17). The RVEDA, RVESA, tricuspid S′ velocity, and TAPSE were assessed according to current guidelines (12). RV FAC, expressed as a percentage, was calculated as (RVEDA-RVESA)/RVEDA. LVEDV, LVESV, and left ventricular ejection fraction (LVEF) were measured using Simpson's biplane method. Speckle tracking was automatically validated using advanced quantification software (EchoPAC Clinical Workstation Software, GE Healthcare) and confirmed visually from 2D images in the apical four-chamber, two-chamber, and three-chamber views. Global and segmental measurements of longitudinal strain were performed by assessing the peak longitudinal strain of the RV free wall. This was calculated as the arithmetic mean of the strain values in the three segments of the ventricular free wall strain obtained from a six-segment region of interest (20, 21).

### Follow-Up and Outcome Measures

The patients were regularly followed up at the outpatient cardiomyopathy clinic. Data including current status, medication use, and re-examination (if necessary), were obtained from clinical visits made regularly or telephone calls to ascertain readmission for worsening. All-cause death was assigned as the primary endpoint and recorded by chart review, telephone contact, and inspection of electronic files for death certificates. For patients without events, the date of the last contact was used for survival analysis. The secondary endpoints were re-hospitalization for cardiac reasons and ICD/cardiac resynchronization therapy (CRT) implantation.

### Statistical Analysis

One-sample Kolmogorov–Smirnov tests and histograms were used to check the normality of the continuous data. Continuous variables were expressed as mean ± SD (for normally distributed variables) or median [interquartile range (IQR)] (for non-normally distributed variables). Levene's test was performed to test the homogeneity of the variances. Normally distributed variables were compared using an unpaired *t*-test (homoscedasticity) or Welch's correction (non-homoscedasticity). Non-normally distributed variables were compared using the Mann–Whitney U test. Categorical data were expressed as percentages and compared using Pearson's χ^2^-test or Fisher's exact test, as appropriate. Survival analysis was performed using the Kaplan-Meier method by defining the time-to-event as the interval from the baseline to the primary endpoint. Kaplan-Meier survival curves were compared using the log-rank test. A univariable Cox proportional hazard model was used to analyze the relationship between the primary endpoint and baseline variables, such as echocardiographic parameters, blood pressure, and serum biochemical parameters. Results were reported as hazard ratios (HRs) with 95% confidence intervals (CIs). Variables with *p* < 0.05, at the univariable analysis or with a prior given clinical relevance were further tested using multivariable Cox regression analysis. Statistical significance was defined as a two-tailed *p*-value of < 0.05. Statistical analysis was performed using GraphPad Prism (Version 8.4.2; GraphPad Software Inc., USA) and SPSS (Version 24.0; SPSS Inc., Chicago, IL, USA).

## Results

### Baseline Characteristics and Outcomes of All Patients

A total of 117 patients with LVNC (mean age: 47.6 ± 18.3 years, 34.2% men) were finally enrolled after excluding two patients with a single ventricle and six with unavailable data. The demographic and baseline characteristics of all subjects are summarized in Table 1. The study population included 64 (54.7%) patients without RV dysfunction and 53 (45.3%) patients with RV dysfunction. Patients with RV dysfunction had lower SBP than the controls (112.3 ± 16.6mmHg vs. 119.2 ± 17.6mmHg, *p* = 0.0437). The RV dysfunction group had higher NT-proBNP (4,506 [1,692, 9,155]pg/ml vs. 603 [98, 2,184]pg/ml, *p* < 0.0001) and Cr (85.0 [72.8, 100.8]μmol/l vs. 72.5 [62.3, 89.8]μmol/l, *p* = 0.0160) than the control group, and no significant difference in the eGFR values was found between the two groups. The baseline echocardiographic parameters are presented in Table 1. The proportion of biventricular involvement was significantly higher in patients with RV dysfunction than in controls (*p* = 0.0155). Patients with RV dysfunction had higher RVEDA than those in the control group (18.6 [15.1, 25.5]cm^2^ vs. 14.8 [11.6, 18.2]cm^2^, *p* < 0.0001) and significantly impaired RV global longitudinal strain (RVGLS) (−8.8 ± 3.8% vs. −17.8 ± 6.0%, *p* < 0.0001). Furthermore, the RV dysfunction group had relatively high LVEDV (170.0 [132.0, 255.5]ml vs. 122.0 [85.0, 155.5]ml, *p* < 0.0001), low LVEF (29.4 ± 13.8% vs. 50.6 ± 14.0%, *p* < 0.0001), and impaired LV global longitudinal strain (LVGLS) (−5.3 [−9.0, −3.2]% vs. −13.9 [−19.6, −10.5]%, *p* < 0.0001). Heart failure medications, including β-blockers, angiotensin-converting enzyme (ACE) inhibitors/angiotensin receptor blockers (ARBs), spironolactone, diuretics, and digoxin, were more frequently used during the follow-up period in the RV dysfunction group than in the controls (all *p* < 0.05).

**Table 1:** Characteristics and outcomes of all patients.

	**All patients (*n* = 117)**	**RV dysfunction (*n* = 53)**	**No RV dysfunction (*n* = 64)**	***P*-value**
Sex				
Men	40 (34.2)	15 (28.3)	25 (39.1)	0.2454
Women	77 (65.8)	38 (71.7)	39 (60.9)	
Age (year)	47.6 ± 18.3	48.1 ± 19.7	47.2 ± 17.3	0.8146
SBP (mmHg)	116.0 ± 17.4	112.3 ± 16.6	119.2 ± 17.6	0.0437
DBP (mmHg)	72.7 ± 13.4	74.3 ± 14.2	71.4 ± 12.7	0.2806
HR (bpm)	80.0 [68.0, 91.0]	82.0 [69.3, 96.0]	78.0 [66.0, 85.5]	0.0899
cTnI (μg/l)	0.030 [0.000, 0.080]	0.034 [0.009, 0.090]	0.016 [0, 0.055]	0.1086
NT-proBNP (pg/ml)	1,818 [297, 6,064]	4,506 [1,692, 9,155]	603 [98, 2,184]	<0.0001
Alb (g/L)	40.8 ± 5.7	40.8 ± 5.6	40.6 ± 5.8	0.8034
Hb (g/L)	138.3 ± 22.2	140.7 ± 19.9	135.8 ± 24.1	0.2657
Cr (μmol/l)	79.5 [66.3, 96.8]	85.0 [72.8, 100.8]	72.5 [62.3, 89.8]	0.0160
eGFR (ml/min/1.73 m^2^)	89.7 ± 30.1	85.1 ± 30.3	93.3 ± 29.7	0.1722
Echocardiographic parameters				
LV and RV involved	35 (29.9)	22 (41.5)	13 (20.3)	0.0155
TAPSE (mm)	17.4 ± 5.2	13.3 ± 3.6	21.1 ± 3.2	<0.0001
S′ (cm/s)	8 [6, 10]	6 [5, 8]	9.6 [9.0, 11.8]	<0.0001
RVEDA (cm^2^)	15.8 [13.1, 20.1]	18.6 [15.1, 25.5]	14.8 [11.6, 18.2]	<0.0001
RVESA (cm^2^)	9.0 [6.5, 13.0]	12.3 [8.6, 18.8]	7.1 [5.3, 9.4]	<0.0001
RV FAC (%)	40.8 ± 14.0	32.1 ± 12.7	49.1 ± 9.8	<0.0001
RVGLS (%)	−13.5 ± 6.8	−8.8 ± 3.8	−17.8 ± 6.0	<0.0001
LVEDV (ml)	142.5 [100.8, 194.5]	170.0 [132.0, 255.5]	122.0 [85.0, 155.5]	<0.0001
LVESV (ml)	87.0 [48.0, 142.0]	129.0 [83.5, 184.0]	60.0 [30.5, 100.5]	<0.0001
LVEF (%)	40.4 ± 17.4	29.4 ± 13.8	50.6 ± 14.0	<0.0001
LVGLS (%)	−10.2 [−14.9, −5.2]	−5.3 [−9.0, −3.2]	−13.9 [−19.6, −10.5]	<0.0001
Follow-up time (months)	69.0 [33.5, 96.0]	49.0 [21.0, 92.0]	82.0 [42.5, 97.8]	0.0747
Heart failure medications				
β-blockers	82 (70.0)	43 (81.1)	39 (61.0)	0.0251
ACE inhibitors/ARB	81 (69.2)	44 (83.0)	37 (58.7)	0.0049
Spironolactone	65 (55.6)	35 (66.0)	30 (46.9)	0.0421
Diuretics	48 (41.0)	29 (54.7)	19 (29.7)	0.0082
Digoxin	37 (31.6)	25 (47.2)	12 (18.8)	0.0013
Outcomes				
All-cause death	18 (15.4)	Hazard ratio: 5.132 [2.003, 13.15]		0.0013
Rehospitalization	35 (29.9)	20 (37.7)	15 (23.4)	0.1075
Implantation ICD/CRT	15 (12.8)	7 (13.2)	8 (12.5)	>0.9999

*Data are shown as mean ± SD or median (interquartile range) for continuous outcomes and as n (%) for categorical outcomes. P-values were based on the unpaired t-test or Mann-Whitney test for continuous outcomes and the Pearson Chi-squared test or the Fisher exact test for categorical outcomes. ACE, angiotensin-converting enzyme; Alb, albumin; ARB, angiotensin receptor blockers; Cr, creatinine; CRT, cardiac resynchronization therapy; cTnI, cardiac troponin I; DBP, diastolic blood pressure; eGFR, estimated glomerular filtration rate; FAC, fractional area change; Hb, hemoglobin; HR, heart rate; ICD, implantable cardioverter-defibrillator; LVEDV, left ventricular end diastolic volume; LVESV, left ventricular end systolic volume; LVEF, left ventricular ejection fraction; LVGLS, LV global longitudinal strain; NT-proBNP, N-terminal fragment of pro-hormone brain natriuretic peptide; RV, right ventricular; RVEDA, right ventricular end-diastolic area; RVESA, right ventricular end systolic area; RVGLS, RV global longitudinal strain; SBP, systolic blood pressure; TAPSE, tricuspid annular plane systolic excursion*.

**Figure 2:**
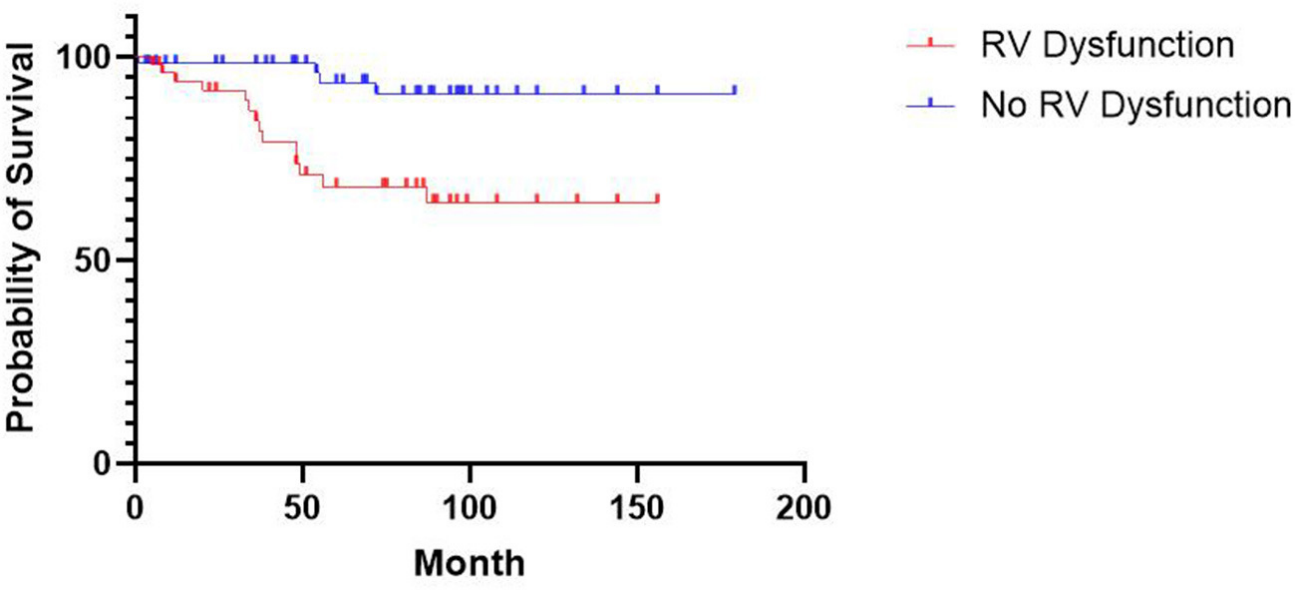
Kaplan-Meier Estimates of Survival According to RV Dysfunction. The difference in survival between patients with and without RV dysfunction was statistically significant (*P* = 0.0013 by the log-rank test).

During a median follow-up time of 69.0 months [33.5, 96.0], 18 patients (15.4%) reached the primary endpoint, including 14 (26.4%) and 4 patients (6.3%) from the RV dysfunction group and the control group, respectively. Patients with RV dysfunction had a higher risk of all-cause mortality than those in the control group (HR, 5.132; 95%CI, 2.003–13.15; *p* = 0.0013). The Kaplan–Meier curve is shown in Figure 2. The relationship between RV dysfunction and heart failure rehospitalization and ICD/CRT implantation was not statistically significant (both *p* > 0.05).

### Adjustment for Confounding Factors

To assess the interaction of RV dysfunction with LV systolic dysfunction on patient prognosis, baseline characteristics and outcomes were analyzed in 72 patients with LVEF < 50%, including 45 (62.5%) and 27 (37.5%) patients with and without RV dysfunction, respectively (Table 2). Higher NT-proBNP (3,070 [1,053, 7,724]pg/ml vs. 1,150 [496, 2,622]pg/ml, *p* = 0.0173), more biventricular involvement (44.4 vs. 7.4%, *p* = 0.0012), and higher RVEDA (18.6 [14.7, 24.9]cm^2^ vs. 15.1 [12.2, 19.3]cm^2^, *p* = 0.0191) were also found in the RV dysfunction group than in the controls. Differences in LVEF also existed between the two groups (27.0 [15.0, 37.0]% vs. 42.0 [32.0, 45.0]%, *p* < 0.0001). However, no significant difference was found in LVEDV (170.0 [132.5, 262.0]ml vs. 152.0 [122.0, 192.0]ml, *p* = 0.1395). In the LV dysfunction population, 12 patients (16.7%) reached the primary endpoint, including 11 (24.4%) from the RV dysfunction group and one patient (3.7%) from the control group. The same results were observed for higher risk of all-cause mortality (HR: 6.582; 95%CI: 2.045–21.19; *p* = 0.0367) in the RV dysfunction group, and no significant difference was observed for heart failure rehospitalization and implantation of ICD/CRT (both *P* > 0.05). Furthermore, Table 3 shows the baseline characteristics of the patients who reached the primary endpoint compared to the rest of the population. The proportion of RV dysfunction was significantly higher in the cardiovascular mortality group than in the control group (77.8% vs. 39.4%, *p* = 0.0039). However, the difference of LVEF (32.4 ± 18.6% vs. 41.6 ± 17.0%, *p* = 0.0629) and LVGLS (−6.6 [−13.0, −4.6]% vs. −10.5 [−15.0, −5.6]%, *p* = 0.1939) between two groups were not statistically significant.

**Table 2:** Characteristics and outcomes of patients with LVEF <50%.

	**RV dysfunction** **(*n* = 45)**	**No RV dysfunction** **(*n* = 27)**	***P*-value**
Sex			
Man	33 (73.3)	17 (63.0)	0.4312
Woman	12 (26.7)	10 (37.0)	
Age (year)	50.2 ± 19.5	51.7 ± 15.7	0.7353
SBP (mmHg)	112.4 ± 17.5	120.5 ± 20.0	0.0910
DBP (mmHg)	74.0 ± 14.0	75.4 ± 13.4	0.6886
HR (bpm)	82.0 [70.0, 96.0]	72.0 [67.5, 81.0]	0.0398
cTnI (μg/l)	0.034 [0.007, 0.090]	0.022 [0.000, 0.085]	0.6648
NT-proBNP (pg/ml)	3,070 [1,053, 7,724]	1,150 [496, 2,622]	0.0173
Alb (g/L)	41.2 ± 4.7	41.0 ± 6.6	0.8801
Hb (g/L)	141.7 ± 17.7	141.0 ± 23.4	0.9035
Cr (μmol/l)	86.0 [73.0, 104.0]	71.0 [59.5, 88.9]	0.0140
eGFR (ml/min/1.73m^2^)	83.2 ± 30.6	94.0 ± 30.8	0.1667
Echocardiographic parameters			
LV and RV involved	20 (44.4)	2 (7.4)	0.0012
TAPSE (mm)	13.3 ± 3.7	20.0 ± 3.0	<0.0001
S′ (cm/s)	6.0 [5.0, 8.0]	9.0 [7.8, 11.0]	<0.0001
RVEDA (cm^2^)	18.6 [14.7, 24.9]	15.1 [12.2, 19.3]	0.0191
RVESA (cm^2^)	12.3 [8.6, 17.9]	7.9 [5.8, 12.3]	0.0003
RV FAC (%)	31.5 [23.2, 41.0]	48.0 [39.0, 57.0]	<0.0001
RVGLS (%)	−8.6 ± 3.8	−14.0 ± 4.2	<0.0001
LVEDV (ml)	170.0 [132.5, 262.0]	152.0 [122.0, 192.0]	0.1395
LVESV (ml)	131.0 [86.5, 198.5]	100.0 [71.0, 118.0]	0.0147
LVEF (%)	27.0 [15.0, 37.0]	42.0 [32.0, 45.0]	<0.0001
LVGLS (%)	−4.9 [−7.8, −3.0]	−10.6 [−12.6, −8.6]	<0.0001
Follow-up time (months)	49.0 [21.0, 92.0]	51.0 [24.0, 96.0]	0.7614
Outcomes			
All-cause death	Hazard ratio: 6.582 [2.045, 21.19]		0.0367
Rehospitalization	17 (37.7)	8 (29.6)	0.6109
Implantation ICD/CRT	7 (15.6)	5 (18.5)	0.7538

*Data are shown as mean ± SD or median (interquartile range) for continuous outcomes and as n (%) for categorical outcomes. P-values were based on the unpaired t-test or Mann-Whitney test for continuous outcomes and Pearson's χ^2^-test or Fisher's exact test for categorical outcomes. ACE, angiotensin-converting enzyme; Alb, albumin; ARB, angiotensin receptor blockers; Cr, creatinine; CRT, cardiac resynchronization therapy; cTnI, cardiac troponin I; DBP, diastolic blood pressure; eGFR, estimated glomerular filtration rate; FAC, fractional area change; Hb, hemoglobin; HR, heart rate; ICD, implantable cardioverter-defibrillator; LVEDV, left ventricular end diastolic volume; LVESV, left ventricular end systolic volume; LVEF, left ventricular ejection fraction; LVGLS, LV global longitudinal strain; NT-proBNP, N-terminal fragment of pro-hormone brain natriuretic peptide; RV, right ventricular; RVEDA, right ventricular end-diastolic area; RVESA, right ventricular end systolic area; RVGLS, RV global longitudinal strain; SBP, systolic blood pressure; TAPSE, tricuspid annular plane systolic excursion*.

**Table 3:** Baseline characteristics of patients reaching the primary endpoint and not reaching the primary endpoint.

	**Patients reaching the primary endpoint** **(*n* = 18)**	**Patients not reaching the primary endpoint (*n* = 99)**	***P*-value**
Sex			
Man	14 (77.8)	63 (63.6)	0.2913
Woman	4 (22.2)	36 (36.4)	
Age (year)	53.0 ± 16.8	46.6 ± 18.5	0.1751
SBP (mmHg)	109.5 ± 13.7	117.2 ± 17.9	0.0931
DBP (mmHg)	65.0 [58.5, 72.5]	72.0 [65.0, 80.0]	0.0464
HR (bpm)	80.0 [66.5, 94.0]	78.0 [68.0, 91.0]	0.7715
cTnI (μg/l)	0.060 [0.025, 0.245]	0.015 [0.000, 0.070]	0.0076
NT-proBNP (pg/ml)	6,185 [2,759, 13,387]	1,150 [182, 3,880]	0.0004
Alb (g/L)	41.4 ± 6.1	41.0 ± 5.7	0.5980
Hb (g/L)	148.0 [111.8, 157.3]	143.0 [121.0, 154.0]	0.9010
Cr (μmol/l)	84.5 [68.3, 110.5]	79.0 [65.8, 92.8]	0.3354
eGFR (ml/min/1.73 m^2^)	85.8 ± 33.4	90.5 ± 29.6	0.5445
Echocardiographic parameters			
LV and RV involved	7 (38.9)	28 (28.3)	0.4064
**RV dysfunction**	**14 (77.8)**	**39 (39.4)**	**0.0039**
TAPSE (mm)	15.4 ± 4.4	17.7 ± 5.3	0.1307
S′ (cm/s)	7.0 [5.1, 8.3]	8.9 [6.8, 10.0]	0.1193
RVEDA (cm^2^)	19.8 [15.7, 22.3]	15.4 [12.3, 19.5]	0.0402
RVESA (cm^2^)	13.7 [10.2, 18.5]	8.5 [6.3, 12.4]	0.0048
RV FAC (%)	30.1 ± 13.0	42.5 ± 13.4	0.0017
RVGLS (%)	−10.7 [−13.6, −5.9]	−13.3 [−8.3, 18.5]	0.0590
LVEDV (ml)	195.0 [145.0, 275.3]	136.5 [96.5, 187.3]	0.0196
LVESV (ml)	136.0 [78.5, 238.3]	86.0 [44.0, 128.8]	0.0340
**LVEF (%)**	**32.4** **±18.6**	**41.6** **±17.0**	**0.0629**
LVGLS (%)	−6.6 [−13.0, −4.6]	−10.5 [−15.0, −5.6]	0.1939

*Alb, albumin; Cr, creatinine; CRT, cardiac resynchronization therapy; cTnI, cardiac troponin I; DBP, diastolic blood pressure; eGFR, estimated glomerular filtration rate; FAC, fractional area change; Hb, hemoglobin; HR, heart rate; ICD, implantable cardioverter-defibrillator; LVEDV, left ventricular end diastolic volume; LVESV, left ventricular end-systolic volume; LVEF, left ventricular ejection fraction; LVGLS, LV global longitudinal strain; NT-proBNP, N-terminal fragment of pro-hormone brain natriuretic peptide; RV, right ventricular; RVEDA, right ventricular end-diastolic area; RVESA, right ventricular end systolic area; RVGLS, RV global longitudinal strain; SBP, systolic blood pressure; TAPSE, tricuspid annular plane systolic excursion. Bold values mean parameters with statistical significance and clinical significance towards outcomes*.

### Predictors of Mortality

Table 4 shows the results of Cox regression analysis of the predictors of the primary endpoint. In the univariable analysis, the primary endpoint was significantly predicted by increased RVEDA, RV dysfunction, impaired RVGLS, and decreased LVEF (all *p* <0.05). However, when variables were introduced into the multivariable Cox regression models, only RV dysfunction (HR: 4.950 [1.378, 17.783], *p* = 0.014) and impaired RVGLS (HR: 1.103 [1.004, 1.212], *p* = 0.041) were identified as independent predictors of mortality, whereas decreased LVEF and increased RVEDA were not (both *p* > 0.05).

**Table 4:** Predictors of mortality.

	**Univariable**		**Multivariable**			
**Predictors**			**Model 1**		**Model 2**	
	**HR (95% Cl)**	***P*-value**	**HR (95% Cl)**	***P*-value**	**HR (95% Cl)**	***P*-value**
LV and RV involved	1.545 [0.598, 3.988]	0.369				
**RVEDA** per 1 cm^2^ increase	**1.060 [1.001, 1.123]**	**0.046**	1.028 [0.960, 1.101]	0.429	1.035 [0.965, 1.110]	0.336
TAPSE <17 mm	2.817 [0.940, 8.443]	0.064				
S′ <10 cm/s	2.104 [0.471, 9.406]	0.330				
RV FAC <35%	3.799 [1.314, 10.986]	0.014				
**RV dysfunction**	**5.158 [1.696, 15.691]**	**0.004**	**4.950 [1.378, 17.783]**	**0.014**		
**RVGLS** per 1% increase	**1.104 [1.004, 1.214]**	**0.042**			**1.103 [1.004, 1.212]**	**0.041**
**LVEF** per 1% increase	**0.966 [0.935, 0.997]**	**0.032**	0.991 [0.952, 1.032]	0.679	0.994 [0.942, 1.048]	0.822
	3.200 [0.892, 11.484]	0.074				
LVGLS per 1% increase	1.068 [0.976, 1.168]	0.154				
SBP (mmHg)	0.975 [0.945, 1.006]	0.116				
DBP (mmHg)	0.978 [0.942, 1.016]	0.256				
cTnI (μg/l)	1.853 [1.134, 3.030]	0.014				
NT-proBNP (pg/ml)	1.000 [1.000, 1.000]	0.006				
Alb (g/L)	1.020 [0.940, 1.107]	0.635				
Hb (g/L)	0.998 [0.978, 1.018]	0.843				
eGFR (ml/min/1.73 m^2^)	0.995 [0.980, 1.010]	0.509				

*Cox proportional hazard regression analysis with univariate and multivariate models for the primary endpoint For both models, a hazard ratio >1 indicated that one category had a higher risk of all-cause death than the reference category, and a hazard ratio <1 indicated that one category had a lower risk of all-cause death than the reference category. Alb, albumin; Cr, creatinine; cTnI, cardiac troponin I; DBP, diastolic blood pressure; eGFR, estimated glomerular filtration rate; FAC, fractional area change; Hb, hemoglobin; LVEDV, left ventricular end-diastolic volume; LVEF, left ventricular ejection fraction; LVGLS, LV global longitudinal strain; NT-proBNP, N-terminal fragment of prohormone brain natriuretic peptide; RV, right ventricular; RVEDA, right ventricular end-diastolic area; RVGLS, RV global longitudinal strain; SBP, systolic blood pressure; TAPSE, tricuspid annular plane systolic excursion. Bold values mean parameters with statistical significance and clinical significance towards outcomes*.

## Discussion

This study confirmed the prognostic value of RV parameters in a relatively large population of patients with LVNC (117 patients) over a median follow-up period of more than 5 years. RV dysfunction is independently associated with all-cause mortality in patients with LVNC, even after correction for LV function. The impaired RVGLS measured by 2D strain imaging indicates early RV systolic function abnormalities and can also predict outcomes in patients with LVNC.

Impaired RV systolic function (defined as RVEF <35% on cardiac MRI) was identified in 50% and 16% of the population, respectively, in two previous researches (10, 11). In this study, RV dysfunction was identified in 45.3% of the patients with LVNC, which is a relatively large proportion.

Regardless of LV failure, RV dysfunction was identified as an independent prognostic marker for LVNC, which might be due to the following reasons.

First, a remarkable RV non-compaction manifestation may indicate serious pathological changes in the myocardium. A substantial relationship between the non-compacted/compacted ratio and changes in global ventricular function has been reported, which may be not just in the LV (22). In our study, morphological biventricular involvement was significantly higher in the RV dysfunction group, accounting for 41.5% of cases. Along with primary myocardial disease, small vessel “dysfunction” with impaired coronary flow reserve and microcirculatory defects causes functional abnormalities (23). Changes in coronary microcirculation affect the development of myocardial fibrosis, which is associated with a poor prognosis (24).

Second, RV dysfunction alone could be an indicator of a poor prognosis in heart failure. Aside from pathological changes, RV dysfunction may be secondary to severe LV failure. In this study, low LVEF, large ventricular size, elevated NT-proBNP, and more frequently use of heart failure medications were observed in the RV dysfunction group. These LV alterations can lead to RV pressure overload (pulmonary arterial hypertension secondary to chronic pulmonary venous hypertension), ventricular interdependence associated with septal dysfunction and limited pericardial flexibility (25, 26), neuro-hormonal interactions, and reduced RV coronary perfusion secondary to decreased systolic driving pressure (27, 28). Further development of RV remodeling and myocardial fibrosis (29) may lead to right heart impairment, thus forming a vicious circle. In a previous cohort of individuals with heart failure with preserved LVEF, significant fluid overload and lower cardiac output were observed in the RV dysfunction subgroup (30). This could result in severe venous congestion and lower SBP, which allows greater requirements for vasoactive medications, all of which contribute to not only increased mortality due to rapid hemodynamic deterioration but also higher rates of acute kidney injury (31, 32). Moreover, lower input (oral uptake) due to gastrointestinal congestion might lead to difficulty in strategies for congestion relief (including monitoring of diuretic administration and/or improvement of organ perfusion), further causing increased mortality (33). Indeed, RV dysfunction is a critical determinant of prognosis in heart failure, regardless of the degree of LV dysfunction (16, 34, 35). This was consistent with the previous finding that decreasing RVEF is independently associated with clinical events including heart failure and death in LVNC, even after adjustment for LVEF (6, 9).

Third, management strategies for right heart failure remain limited. In addition to the use of diuretics to relieve symptoms, effective ways to improve the histological changes of the right ventricle are lacking. Simultaneously, the influence of left heart failure on prognosis has not been observed in this study, mainly because of the good management of left heart failure. Patients in the present cohort were regularly followed up in our clinical center, and medications including β-blockers, ACE inhibitors/ARBs, spironolactone, diuretics, and digoxin were used following current international guidelines (36). This is the reason why RV dysfunction was associated with mortality in LVNC after correction of LV function, for better management of LV dysfunction in clinical cases.

In this study, the evidence obtained for heart failure rehospitalization and implantation of ICD/CRT was weak. Readmissions were mainly due to worsening symptoms including dyspnea. It is common for left-sided heart failure to receive more clinical attention, whereas signs of systemic congestion (such as edema) are prone to be ignored unless the condition is severe. ICD implantation mainly targets malignant arrhythmia to prevent sudden death. Likewise, CRT mainly indicates non-synchronized ventricular contraction caused by the left bundle branch block, which may not be relevant to RV dysfunction. Additionally, few patients choose to accept device therapies for financial reasons.

RV function is not easily obtained and is a time-consuming procedure because of the complex geometry of the RV and the lack of specific right-sided anatomic landmarks to be used as reference points (12). To date, RV assessment is not as systematic as left heart evaluation and is prone to be ignored by clinicians. In this study, the prognostic value of RV dysfunction in LVNC has been highlighted, which indicates the need to emphasize the evaluation of RV function. Moreover, RVGLS was also found to have prognostic value since it detects subtle changes in RV function in several populations (37, 38) and provides early hints during the preclinical stage. In line with the current guidelines, RV function is considered to be of general prognostic importance in heart failure and quantitative RV assessment appears mandatory (39).

This study has some limitations. This was a single-center study with 117 patients with LVNC included and the right ventricle was not optimally visualized in all cases, resulting in missing data on RV function parameters, such as tricuspid S′. Speckle tracking requires user experience and high-quality images, which are not currently recommended for routine RV assessment. Therefore, large sample-sized studies with long-term follow-up are required to confirm this association in patients with LVNC.

## Conclusion

This study demonstrated that RV dysfunction is a strong independent and incremental risk factor for all-cause mortality in patients with LVNC. Two-dimensional strain imaging by STE seems to be a quantitative tool for early RV systolic function abnormalities and is associated with outcomes in patients with LVNC. This finding may have implications for the risk assessment of patients with LVNC, suggesting a regular and quantitative assessment of RV function in patients with LVNC.

## Data Availability Statement

The raw data supporting the conclusions of this article will be made available by the authors, without undue reservation.

## Ethics Statement

The studies involving human participants were reviewed and approved by the Ethical Committee of Peking Union Medical College Hospital, Chinese Academy of Medical Science. Written informed consent to participate in this study was provided by the participants' legal guardian/next of kin.

## Author Contributions

WW was responsible for data screening, data extraction and analysis, and writing of the manuscript. WC, XL, and LF were responsible for the echocardiography data and checked and reviewed the final manuscript. All authors contributed to the article and approved the submitted version.

## Funding

This work was sponsored by the Chinese National Natural Science Foundation (Grant Number 81670349) and the Beijing Natural Science Foundation (Grant Number 7172166).

## Conflict of Interest

The authors declare that the research was conducted in the absence of any commercial or financial relationships that could be construed as a potential conflict of interest.

## Publisher's Note

All claims expressed in this article are solely those of the authors and do not necessarily represent those of their affiliated organizations, or those of the publisher, the editors and the reviewers. Any product that may be evaluated in this article, or claim that may be made by its manufacturer, is not guaranteed or endorsed by the publisher.

## Abbreviations

ACE: angiotensin-converting enzyme
ARB: angiotensin receptor blocker
Cr: creatinine
CRT: cardiac resynchronization therapy
cTnI: cardiac troponin I
DBP: diastolic blood pressure
NT-proBNP: N-terminal fragment of pro-hormone brain natriuretic peptide
FAC: fractional area change
eGFR: estimated glomerular filtration rate
HR: heart rate
ICD: implantable cardioverter-defibrillator
LV: left ventricle
LVEDV: left ventricular end diastolic volume
LVESV: left ventricular end systolic volume
LVEF: left ventricular ejection fraction
LVGLS: left ventricular global longitudinal strain
LVNC: left ventricular non-compaction cardiomyopathy
RV: right ventricular
RVEDA: right ventricular end diastolic area
RVESA: right ventricular end systolic area
RVGLS: right ventricular global longitudinal strain
SBP: systolic blood pressure
TAPSE: tricuspid annular plane systolic excursion
2D: two-dimensional.
